# Clinical characteristics and individualized treatment strategies for children with type 1 diabetes and exogenous insulin antibody syndrome: a retrospective case series and literature review

**DOI:** 10.3389/fendo.2026.1770560

**Published:** 2026-03-26

**Authors:** Liang Zhang, Xinyuan Shen, Lulu Cui, Ying Zhang, Jiaoru Yang, Wenxin Liu, Lili Wang, Sheng Guo

**Affiliations:** Department of Endocrine and Metabolism, Shanghai Children’s Hospital, School of medicine, Shanghai Jiao Tong University, Shanghai, China

**Keywords:** continuous glucose monitoring, exogenous insulin antibody syndrome, glycemic variability, mycophenolate mofetil, personalized treatment, type 1 diabetes

## Abstract

**Objective:**

This study investigates the clinical characteristics, diagnostic strategies, and therapeutic outcomes of individualized treatment for children with type 1 diabetes mellitus (T1DM) complicated by exogenous insulin antibody syndrome (EIAS).

**Methods:**

A retrospective case series study was conducted on five pediatric patients diagnosed with T1DM+EIAS at a single center between January 2016 and January 2026. Among 1,245 T1DM patients evaluated, 5 (0.4%) met EIAS diagnostic criteria. Data included demographics, clinical manifestations, laboratory findings (insulin antibodies [IA], C-peptide, continuous glucose monitoring [CGM]), treatment regimens, and outcomes. Longitudinal autoantibody profiles, thyroid function, immune parameters, and cytokines were assessed at four time points. A narrative review of published EIAS cases was conducted following PRISMA guidelines.

**Results:**

Case 1 achieved glycemic stability using ultra-rapid insulin with closed-loop pump (6-hour active insulin duration). Case 2, a 1-year-old infant, required regular insulin every 4 hours (six doses daily). Case 3 switched from pump to conventional multiple daily injections (MDI) with 4–6 daily injections. Case 4 used 4–6 daily aspart doses plus once-daily glargine. All four achieved TIR >70%. Case 5, with refractory disease and IA titer of 33.40 COI, received mycophenolate mofetil (MMF) 600–1,000 mg/m²/day. After 3 months, TIR improved to >70%, TBR <5%, and IA titers decreased by >30%. MMF discontinuation resulted in rapid recurrence of instability within 4 weeks.

**Conclusion:**

EIAS is a rare cause of severe glycemic dysregulation in pediatric T1DM. CGM metrics (TIR, TBR, CV) are essential for assessment, as HbA1c may not reflect glycemic variability. Individualized insulin optimization improves TIR in most patients. For refractory cases, MMF may offer a potential therapeutic option. Due to the observational nature, small sample size (n=5), absence of a control group, and lack of blinding, these findings should be considered hypothesis-generating. Causal inferences cannot be drawn, and the results require validation in prospective, multicenter, controlled studies.

## 1 Introduction

Type 1 diabetes (T1DM) is one of the most common endocrine metabolic disorders in childhood. Its core pathophysiological change involves the destruction of pancreatic beta cells by the immune system, leading to absolute insulin deficiency (1). Exogenous insulin replacement therapy is the cornerstone for sustaining life and controlling blood glucose in children with T1DM. However, during treatment, some children develop a rare yet highly challenging complication—exogenous insulin antibody syndrome (EIAS) (2). EIAS occurs when the body produces high-titer antibodies against exogenous insulin. These antibodies bind to injected insulin, forming an unstable reservoir of insulin-antibody complexes. This reservoir randomly dissociates, releasing active insulin and fundamentally altering insulin pharmacokinetics and pharmacodynamics (3). Clinically, this manifests as severe, unpredictable fluctuations in blood glucose levels between extremely high (when insulin is bound and unable to function) and extremely low (when the complex dissociates, causing concentrated insulin release). This significantly impacts the child’s quality of life, increases the risk of acute complications, and poses a threat to long-term prognosis (4).

Currently, there is no unified diagnostic standard for EIAS, which is typically determined through a comprehensive assessment of characteristic clinical manifestations, detection of high-titer insulin antibodies (IA), and poor response to conventional insulin regimen adjustments (5). It is important to note that IA in EIAS refers to antibodies against exogenous insulin, distinct from insulin autoantibodies (IAA) that are used as predictive markers for T1DM development. This distinction is clinically significant, as the presence of IAA at T1DM diagnosis is expected, whereas IA formation against exogenous insulin represents a pathological complication.

Treatment strategies for EIAS lack high-level evidence-based medical support, with current approaches primarily consisting of empirical therapies (6). These include switching insulin formulations, modifying administration methods, and attempting immunosuppressive therapy in refractory cases. Mycophenolate mofetil (MMF), a selective inhibitor of inosine monophosphate dehydrogenase (IMPDH), suppresses T-cell and B-cell proliferation, thereby reducing antibody production (7, 8). It has demonstrated favorable outcomes in treating multiple autoimmune diseases.

Recent advances in our understanding of EIAS pathophysiology have highlighted significant knowledge gaps in the pediatric population. Hu and Chen (2018) (6) first systematically described the clinical characteristics of EIAS, but their study primarily focused on adult patients with type 2 diabetes. Saba et al. (2024) (4) reported the first successful use of MMF in a pediatric patient with EIAS, yet systematic treatment protocols remain undefined. Currently, diagnostic criteria, optimal timing for immunosuppressive therapy, and long-term safety profiles for MMF in pediatric EIAS are not established.

This retrospective case series was designed to address these knowledge gaps by characterizing the clinical features, treatment responses, and outcomes of pediatric patients with T1DM complicated by EIAS. The specific objectives were (1): to characterize the clinical and laboratory features of EIAS in children with T1DM (2); to evaluate the association between individualized insulin regimen adjustments and glycemic outcomes; and (3) to assess the efficacy and safety of MMF as second-line therapy in refractory cases. Given the rarity of EIAS in pediatric T1DM (estimated incidence <0.5%) and the urgent need for treatment during acute episodes, a randomized controlled trial was neither ethically feasible nor practically achievable. Therefore, we employed a retrospective case series design, which represents the most commonly used observational study design for rare diseases. This approach allows for detailed characterization of clinical features, treatment responses, and prognostic factors, providing hypothesis-generating data for future prospective studies.

## 2 Materials and methods

### 2.1 Study design and ethical approval

This retrospective case series study was conducted at the Department of Endocrinology and Metabolism, Shanghai Children’s Hospital. The study was reviewed and approved by the Ethics Review Committee of Shanghai Children’s Hospital (No. 2023R018-E01). Written informed consent to participate in this study was provided by the participants’ legal guardians.

### 2.2 Study population and sample size

Between January 2016 and January 2026, a total of 1,245 pediatric patients with T1DM were evaluated at our center. Among these, 5 patients (0.4%) met the diagnostic criteria for EIAS and were included in this study. The sample size was determined by the number of eligible patients presenting during the study period, given the rarity of EIAS in pediatric T1DM. According to guidelines for rare disease research, case series with 5 or more patients can provide valuable preliminary evidence when effect sizes are large and consistent across cases (9). Our sample of 5 patients represents approximately 22% of all reported pediatric T1DM+EIAS cases (n~25), maximizing the available evidence for this rare condition.

Patient Selection Flow: Total diabetic cohort (N = 1,409) → T1DM patients assessed (n=1,245) → Patients with severe glycemic variability (n=35) → Patients undergoing EIAS screening (n=22) → Patients fulfilling EIAS diagnostic criteria (n=5) → Final analytical cohort (n=5).

### 2.3 Inclusion and exclusion criteria

Inclusion Criteria (1): Meeting the diagnostic criteria for T1DM published by the International Society for Pediatric and Adolescent Diabetes (ISPAD) (10) (2); Clinical presentation of persistent (>1 month) severe glycemic fluctuations unresponsive to conventional insulin regimen adjustments, characterized by frequent alternating episodes of hyperglycemia (>16.7 mmol/L) and unpredictable severe hypoglycemia (<3.0 mmol/L) (3); High-titer positive serum insulin antibodies (IA ≥1.0 COI; high-titer defined as ≥1.5 COI). The assay’s cutoff value, as determined by the manufacturer (Shenzhen YHLO Biotech Co., Ltd., Shenzhen, China), is 1.0 COI. Values ≥1.0 are considered positive, indicating elevated antibody levels. Values ≥1.5 COI are considered high-titer, indicating a strong antibody response (4); Extremely low or undetectable C-peptide levels, indicating failure of endogenous insulin secretion (5); Exclusion of other factors potentially causing blood glucose fluctuations, such as improper insulin injection technique, irregular diet, infection, adrenal insufficiency, or thyroid dysfunction.

Exclusion Criteria (1): Incomplete clinical data preventing comprehensive assessment (2); Concurrent systemic diseases significantly affecting glucose metabolism (e.g., Cushing syndrome, growth hormone deficiency) (3); History of insulinoma or other causes of hypoglycemia (4); Concurrent use of medications known to affect glucose metabolism (e.g., corticosteroids, atypical antipsychotics).

Differential Diagnosis: Alternative causes of glycemic variability were systematically excluded: (a) Insulinoma (fasting insulin levels typically <1,000 μIU/mL versus levels sometimes >1,000 μIU/mL in EIAS; negative imaging; negative sulfonylurea screen); (b) Type B insulin resistance (insulin receptor antibodies, acanthosis nigricans, normal or elevated insulin levels with severe resistance); (c) Surreptitious insulin use (low C-peptide, negative sulfonylurea screen, characteristic clinical history); (d) Counter-regulatory hormone deficiencies (normal cortisol, growth hormone, and thyroid function); and (e) Drug-induced insulin autoimmune syndrome (no exposure to sulfhydryl-containing medications such as methimazole, thiamazole, carbimazole, α-lipoic acid, captopril, clopidogrel, penicillamine, or proton pump inhibitors).

### 2.4 Antibody detection methods

Insulin antibodies and islet cell antibodies were measured using direct chemiluminescence immunoassay (CLIA), a two-step indirect immunoassay performed on a fully automated analyzer. The resulting chemiluminescence (measured as relative light units, RLU) is proportional to the amount of antibodies in the sample. The instrument automatically compares the sample RLU with the calibration curve to calculate the Cut-Off Index (COI).

Reference ranges and interpretation: IA and ICA: expressed as COI (<0.9 negative; ≥1.0 positive; ≥1.5 high-titer); GADA and IA-2A: expressed in U/mL; ZnT8A: expressed in AU/mL; (0.00-10.00 reference range; >10.0 U/mL positive).

### 2.5 Longitudinal assessments

To characterize the temporal evolution of EIAS and exclude alternative autoimmune etiologies, we performed comprehensive longitudinal assessments at four time points (1): T1DM disease onset (2), EIAS diagnosis (3), 6 months post-EIAS treatment, and (4) 1 year post-EIAS treatment. Analyses included: (i) diabetes-related autoantibodies (IA, GADA, IA-2A, ICA, ZnT8A) by chemiluminescence immunoassay; (ii) thyroid function and autoantibodies (TSH, FT3, FT4, TPOAb, TgAb, TRAb); (iii) systemic immune parameters (immunoglobulins IgG/IgA/IgM/IgE, complement C3/C4, ANA); and (iv) serum cytokine profiles (IL-1β, IL-2, IL-4, IL-5, IL-6, IL-8, IL-10, IL-12p70, IL-17, IFN-α, IFN-γ, TNF-α) by flow cytometry (**Supplementary Tables 1**-**4**).

### 2.6 CGM data analysis

Continuous glucose monitoring (CGM) data were collected using certified devices (Abbott FreeStyle Libre, Sibionic, Medtronic Guardian) over a minimum 14-day monitoring period. Data quality requirements included (1): >70% valid glucose readings (2); calibration within manufacturer specifications (3); no gaps >2 hours in continuous monitoring.

CGM metrics were calculated according to international consensus guidelines (11): Time in Range (TIR): Percentage of time glucose was within 3.9-10.0 mmol/L (70–180 mg/dL); Time Below Range (TBR): Percentage of time glucose was <3.9 mmol/L (<70 mg/dL); Time Above Range (TAR): Percentage of time glucose was >10.0 mmol/L (>180 mg/dL); Coefficient of Variation (CV): (Standard deviation of glucose/Mean glucose) × 100%.

Target CGM goals for pediatric T1DM according to ISPAD 2024 guidelines (10): TIR >70%, TBR <4% (<3.9 mmol/L), TBR <3.0 mmol/L <1%, CV ≤36%.

### 2.7 Statistical analysis

Given the small sample size (n=5) and the exploratory nature of this case series, we employed descriptive statistical methods only. NO INFERENTIAL STATISTICAL TESTS WERE PERFORMED, as the limited sample size precludes meaningful hypothesis testing and could lead to misleading conclusions. All data are presented for descriptive purposes only, and mean values should not be interpreted as representative of a larger population.

Continuous variables were summarized using descriptive statistics: normally distributed variables were expressed as mean ± standard deviation (M ± SD), while non-normally distributed variables were expressed as median (interquartile range) [M (IQR)]. Categorical variables were expressed as frequency (percentage). Individual patient data are presented to allow readers to assess the consistency and magnitude of observed changes.

Data organization and descriptive analysis were performed using GraphPad Prism version 9.3.1 (GraphPad Software, San Diego, CA, USA). The analysis code and de-identified dataset are available from the corresponding author upon reasonable request.

## 3 Results

### 3.1 Baseline characteristics

A total of five pediatric patients (three males and two females) were included. The median age at T1DM diagnosis was 3.0 years (range 0.6–6.9), and the median age at EIAS diagnosis was 4.9 years (range 3.0–7.9), yielding a median interval of 2.4 years (range 1.0–3.8) between T1DM onset and the emergence of typical EIAS symptoms.

**Figure 1:**
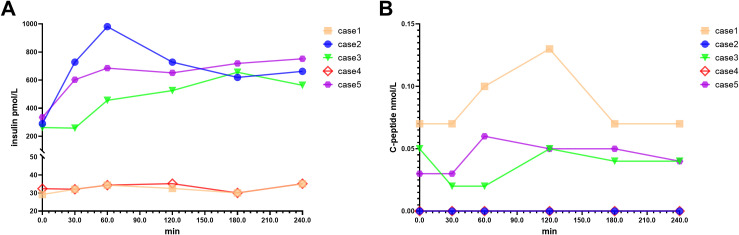
Insulin and C−peptide metabolism profiles in pediatric EIAS patients (n=5). **(A)** Plasma insulin concentrations showing marked interindividual variability. Baseline levels ranged from 29.3–334.8 pmol/L, diverging into two distinct patterns: Cases 2, 3, and 5 exhibited sustained hyperinsulinemia (290–981 pmol/L) peaking at 60–180 minutes, whereas Cases 1 and 4 maintained consistently levels (~30–35 pmol/L). **(B)** Corresponding C-peptide concentrations demonstrating severely suppressed endogenous secretion (<0.003–0.13 nmol/L) without glucose-stimulated increment. The marked dissociation between elevated circulating insulin and minimal C-peptide is consistent with EIAS. DATA ARE PRESENTED FOR DESCRIPTIVE PURPOSES ONLY. Individual trajectories shown as colored lines. NO INFERENTIAL STATISTICAL COMPARISONS WERE PERFORMED due to limited sample size (n=5).

At EIAS diagnosis, patients 2 and 4 had fasting and postprandial C-peptide levels below the lower limit of detection (<0.003 nmol/L), while the remaining patients showed extremely low C-peptide concentrations (0.03-0.13 nmol/L), indicating a complete loss of endogenous insulin secretion (**Figure 1B**). The insulin metabolic curve was markedly prolonged, with no significant decline observed until 240 minutes after a bolus insulin injection, suggesting that exogenous insulin was not metabolized normally (**Figure 1A**).

All patients exhibited typical clinical manifestations for at least one month before EIAS diagnosis: daily blood glucose levels fluctuated dramatically between 2.5 mmol/L and 28 mmol/L, with frequent, unexplained severe hypoglycemic episodes—particularly at night or several hours post-prandially—accompanied by persistent hyperglycemia and a marked increase in daily insulin requirements. Baseline IA titers were elevated in all five patients (**Table 1**).

**Table 1:** Baseline clinical characteristics and laboratory findings in 5 children with T1DM and EIAS.

Case	Gender	Age at T1DM Diagnosis (years)	Age at EIAS Diagnosis (years)	Islet autoantibody profile					Baseline HbA1c (mmol/mol [%])
				IA (<0.9 COI)	IA-2A (0.00~10.00 U/mL)	GADA (0.00~10.00 U/mL)	ICA (negative)	ZnT8A (0.00~10.00 AU/mL)	
1	Female	6.9	7.9	1.58 ↑	52.6↑	610.57 ↑	negative	0.00	43.2 [6.1]
2	Female	1.0	3.7	5.22 ↑	negative	3.33	1.18 ↑	0.00	56.3 [7.3]
3	Male	3.3	4.9	2.66 ↑	negative	<1.0	negative	negative	73.8 [8.9]
4	Male	3.0	6.8	2.16 ↑	negative	14.55 ↑	3.17 ↑	negative	116.4 [12.8]
5	Male	0.6	3.0	33.40 ↑	negative	13.10 ↑	5.93 ↑	0.11	149.2 [15.8]

IA, Insulin Autoantibody; IA-2A, Tyrosine Phosphatase Antibody; GADA, Glutamic Acid Decarboxylase Antibody; ICA, Islet Cell Antibody; ZnT8A, Zinc Transporter 8 Antibody. IA and ICA values are expressed as COI (Cut-Off Index). The assay’s cutoff value, as determined by the manufacturer, is 1.0 COI. Values ≥ 1.0 are considered positive, indicating elevated antibody levels. Values ≥ 1.5 COI are considered high-titer, indicating a strong antibody response. IA-2A and GADA values are expressed in U/mL, ZnT8A values are expressed in AU/mL with a positivity threshold of > 10 as per the assay’s specifications. IA-2A and GADA values >10.0 U/mL are considered strongly positive. The upper limit of the reference range (10.0 U/mL) represents the threshold for positivity; values exceeding this threshold indicate high-titer positivity. The symbol "↑" indicates values exceeding the reference range upper limit or positivity threshold.

### 3.2 Longitudinal evolution of diabetes-related autoantibodies and immune markers

Longitudinal monitoring revealed dynamic fluctuations in IA titers across all five patients from disease onset through 1-year post-EIAS diagnosis (**Supplementary Table 1**):

- Case 1: IA converted from negative at T1DM onset to 1.58 COI at EIAS diagnosis, peaked at 2.56 COI at 6 months post-treatment, then stabilized at 1.56 COI at 1 year.
- Case 2: IA increased progressively from 0.69 COI at disease onset to 5.22 COI at EIAS diagnosis, then declined to 3.66 COI (6 months) and 2.66 COI (1 year) following insulin regimen optimization.
- Case 3: IA emerged from negative at disease onset to 2.66 COI at EIAS diagnosis, with minimal change at 6 months (2.68 COI).
- Case 4: IA showed a notable 13.5-fold increase from 0.16 COI at T1DM onset (April 2020) to 2.16 COI at EIAS diagnosis (November 2023), then decreased to 0.26 COI at 6 months post-intervention before rising again to 3.17 COI at 1 year, demonstrating the fluctuating nature of antibody-mediated insulin resistance.
- Case 5: IA increased dramatically from 0.22 COI at disease onset to 33.40 COI at EIAS diagnosis, then declined progressively to 21.4 COI (6 months) and 13.75 COI (1 year) following MMF therapy.

Parallel longitudinal assessments of thyroid function (**Supplementary Table 2**), systemic immune parameters (**Supplementary Table 3**), and inflammatory cytokines (**Supplementary Table 4**) revealed distinct immunological trajectories across patients. Thyroid autoimmunity (elevated TPOAb) developed in Case 1 at 1-year follow-up (95.5 IU/mL), while Case 4 showed cytokine elevation (IFN-γ 31.01 pg/mL, IL-8 25.03 pg/mL) at 1-year follow-up. These findings suggest heterogeneous autoimmune profiles among patients with EIAS.

### 3.3 Individualized treatment strategies and outcomes

#### 3.3.1 Insulin regimen optimization (cases 1-4)

To address the core pathophysiology of EIAS—abnormal insulin pharmacokinetics—we refined insulin regimens for four children. The overarching aim was to emulate physiological insulin secretion while reducing the “antibody reservoir” effect that arises from high-dose single injections.

Key strategies applied (1): Switching insulin formulations—long-acting analogues were replaced with faster-acting, peak-defined short-acting human insulin or ultra-rapid analogues (e.g., insulin aspart, insulin lispro). Rapid-acting preparations bind antibodies for a shorter duration, yielding more predictable action curves (12) (2). Increasing dosing frequency—for those on MDI, the schedule was expanded from four to six-to-eight injections per day, delivering smaller doses per injection (3). Optimizing pump continuous subcutaneous insulin infusion (CSII) settings—for pump users, basal rates were fine-tuned according to CGM data, and pre-meal bolus modes were changed to extended bolus formats: square-wave bolus (the entire meal bolus delivered evenly over an extended period, typically 3–6 hours) or dual-wave bolus (a portion of the bolus delivered immediately as a standard bolus, with the remainder delivered as an extended square-wave over 3–6 hours). This simulated a gradual intravenous infusion, limiting local high-concentration spikes that promote rapid antibody binding and improving insulin absorption (13, 14).

##### 3.3.1.1 Case 1: 6-year-11-month-old female

Initial presentation (21 Oct 2022): Polyuria, polydipsia, weight loss, vomiting; DKA (pH 7.10, PCO_2_ 2.00 kPa, PO_2_ 10.50 kPa, glucose 28 mmol/L). Managed in PICU with fasting, fluid resuscitation, IV insulin. Transferred to ward (22 Oct) for diabetes education, dietary control, CGM, and subcutaneous insulin aspart/glargine. Discharged on total daily dose 0.80 U/kg (46% glargine) with CGM TIR 82%.

Readmission (1 Oct 2023): Marked glucose variability (2.8–22 mmol/L) and recurrent hypoglycemia with impaired consciousness despite outpatient regimen and pump use. Positive insulin-autoantibody test (IA 1.58 COI) confirmed EIAS.

Intervention: Switched to ultra-rapid insulin (Lyumjev, lispro) and programmed the closed-loop pump with a 6-hour active insulin duration, embodying the square-wave bolus principle. Basal rates were refined using CGM data.

Outcome: At discharge, pre-prandial glucose 4.0–9.0 mmol/L, post-prandial 5.4–7.7 mmol/L; basal insulin 14.2 U/24 h; average bolus doses 6 U (breakfast), 5 U (lunch), 6 U (dinner). No further severe hypoglycemia.

##### 3.3.1.2 Case 2: 1-year-old female infant

The patient was 1 year old at initial T1DM diagnosis (January 2021) and approximately 3.7 years old (3 years, 8 months) at the time of EIAS diagnosis and readmission (September 2023), representing an interval of approximately 2.7 years between T1DM onset and EIAS development.

Initial presentation (10 Jan 2021): Polyphagia, polyuria, poor appetite, lethargy; DKA (pH 7.12, PCO_2_ 21.29 kPa, glucose 27.1 mmol/L) with moderate dehydration. PICU treatment with fluids and IV insulin (0.1–0.05 U/kg·h) lowered glucose to 8–15 mmol/L. Transferred to ward on diabetic diet with titrated subcutaneous regular human insulin and NPH insulin. Discharged with pre-meal glucose 6.4–7.1 mmol/L, post-prandial 8.5–11.3 mmol/L, bedtime 5.4–6.8 mmol/L.

Readmission (17 Sep 2023): Severe glycemic variability, alternating hypo-/hyperglycemia, and a seizure-inducing hypoglycemia (2.5 mmol/L). Despite insulin adjustments, control remained poor. Insulin-autoantibody test positive (5.22 COI) confirmed EIAS.

Intervention: Implemented a regimen of regular insulin administered subcutaneously every 4 hours (total of six doses per day), reflecting the increased dosing frequency and smaller per-dose volume strategy.

Outcome: At discharge, fasting glucose 5.3–7.3 mmol/L, post-prandial 7.2–11.9 mmol/L, with no further severe hypoglycemic episodes.

##### 3.3.1.3 Case 3: 4-year-11-month-old male

Initial presentation (31 Aug 2024, age 4 yr 11 mo): Six-hour vomiting, mild dehydration, intestinal infection, and diabetic ketoacidosis (DKA: pH 7.13, PCO_2_ 3.9 kPa, HCO_3_^−^ 9.6 mmol/L, glucose 27.9 mmol/L). Notably, the patient had been diagnosed with type 1 diabetes in December 2022 (approximately 20 months prior to this admission), though detailed prior treatment records were unavailable. He was treated with ceftriaxone, intravenous insulin, and fluid resuscitation, then transferred to the ward for diabetic dietary management, continuous glucose monitoring, and insulin-pump therapy.

Course and Diagnosis: Despite pump therapy, the patient exhibited persistent daytime hyperglycemia, nocturnal hypoglycemia, and delayed, prolonged insulin-release profiles consistent with insulin-antibody mediated clearance impairment. Laboratory confirmation revealed positive insulin autoantibodies (IA 2.66 COI), establishing the diagnosis of EIAS superimposed on pre-existing type 1 diabetes.

Intervention: Transitioned from pump therapy to conventional MDI per parental preference, utilizing insulin aspart (rapid-acting analog) administered 4–6 times daily, with a total dose of 0.875 U/kg/day.

Outcome: No severe hypoglycemic episodes occurred following the regimen switch; glycemic variability improved with resolution of nocturnal hypoglycemia.

##### 3.3.1.4 Case 4: 3-year-1-month-old male

Initial presentation (2 Apr 2020, age 3 yr 1 mo): Polyphagia, polydipsia, polyuria, weight loss, DKA, moderate dehydration, hypokalemia, growth retardation, bronchitis. Labs: pH 7.09, glucose 26.7 mmol/L, C-peptide 0.06 nmol/L, GAD-65 antibody 14.55 IU/mL, IA 0.16 COI, islet-cell antibody 3.17 COI. Latest admission (12 Jan 2025, age 7 yr 10 mo): Poor control, EIAS (diagnosed in Nov 2023 with IA 2.16 COI), vitamin D deficiency.

Notably, following intensive insulin optimization, IA transiently decreased to 0.26 COI at 6 months, but rebounded to 3.17 COI at 1-year follow-up, suggesting ongoing immune activation despite clinical stabilization (**Supplementary Table 1**).

Intervention: Adopted a regimen of 4–6 daily aspart insulin doses plus once-daily glargine, reflecting both the switch to faster-acting formulations and increased injection frequency.

Outcome: Discharged on 26 U total (~0.92 U/kg/day) with no severe hypoglycemia.

These results demonstrate that, by understanding the underlying pathophysiology of pediatric EIAS and applying targeted insulin-pharmacokinetic optimizations—formulation switches, higher dosing frequency, and precise pump adjustments—stable glycemic control can be achieved in most affected children (15).

#### 3.3.2 Immunosuppressive therapy for refractory EIAS (case 5)

##### 3.3.2.1 Case 5: infantile-onset T1DM with Klinefelter syndrome

This male infant, from a rural area of Jiangxi Province, China, presented with classic symptoms of diabetes mellitus (polydipsia, polyuria, weight loss) and diabetic ketoacidosis at 8 months of chronological age (diagnosed in 2021). Notably, retrospective history indicated that symptomatic onset likely occurred before 6 months of age, initially raising strong clinical suspicion for neonatal diabetes mellitus (NDM). However, comprehensive genetic analysis including whole-exome sequencing and karyotyping revealed no pathogenic variants in monogenic diabetes genes (including *KCNJ11, ABCC8, INS*, etc.). Instead, a 47,XXY karyotype consistent with Klinefelter syndrome was identified, along with a *de novo* duplication of Xp22.33-q28 (~155.04 Mb) that was absent in both parents. Given the negative monogenic diabetes panel and the presence of high-titer islet autoantibodies (IA 33.40 COI, GADA 13.10 U/mL, ICA 5.93 COI), the final diagnosis was infantile-onset type 1 diabetes mellitus rather than NDM. The patient also exhibited marked global developmental delay.

From diagnosis onward, glycemic control proved exceptionally difficult despite multiple insulin regimens. Fasting glucose values fluctuated between 2.7 mmol/L and 15.8 mmol/L, with frequent hospitalizations for severe hypoglycemia, hyperglycemia, and ketoacidosis. Serial C-peptide measurements remained extremely low (0.04 nmol/L), indicating minimal endogenous insulin secretion. In 2023, persistent instability and high insulin requirements prompted suspicion of EIAS. An insulin release test demonstrated markedly elevated insulin concentrations (334.8 pmol/L at 0 min) in the setting of low C-peptide, a classic pattern for EIAS. CGM revealed extreme variability (CV 43.5%), with TIR remaining <40% despite intensive insulin-pump therapy, extended pre-meal bolus timing, and attempts to achieve a 6-hour insulin action profile. IA titers remained persistently elevated (>25 COI), suggesting a high-volume, low-affinity antibody pool that overwhelmed pharmacokinetic adjustments.

In June 2024, immunosuppressive therapy with mycophenolate mofetil (MMF) was initiated at 150 mg twice daily, escalated to 600–1,000 mg/m²/day over two weeks, alongside an adjusted insulin regimen (insulin degludec plus insulin aspart).

Outcomes:

At 1 month: Daily insulin requirement began to decline and glucose variability decreased.

At 3 months: IA titers fell by approximately 50% from baseline (from >30 COI to ~16 COI); TIR stabilized at >70%; TBR fell below 5%; and no further severe hypoglycemia requiring medical intervention occurred. HbA1c improved from 149.2 mmol/mol (15.8%) at baseline to 62 mmol/mol (7.8%).

At 6 months: At the parents’ request, MMF was discontinued. Within 4 weeks of cessation, the patient experienced rapid recurrence of severe glycemic instability, with TIR falling to <50% and renewed episodes of nocturnal hypoglycemia. IA titers began to rise again.

Re-treatment: MMF was promptly re-initiated at the previous dose (1,000 mg/m²/day), with restoration of glycemic stability within 2 weeks. This episode confirmed the necessity of ongoing immunosuppressive therapy to maintain antibody suppression and metabolic control.

Safety Monitoring: Throughout MMF therapy, complete blood count, liver and renal panels were monitored every 2–4 weeks, and immunoglobulin levels every 3 months. No clinically significant bone-marrow suppression, hepatic/renal toxicity, or severe infections were observed. Mild gastrointestinal discomfort resolved spontaneously.

**Figure 2:**
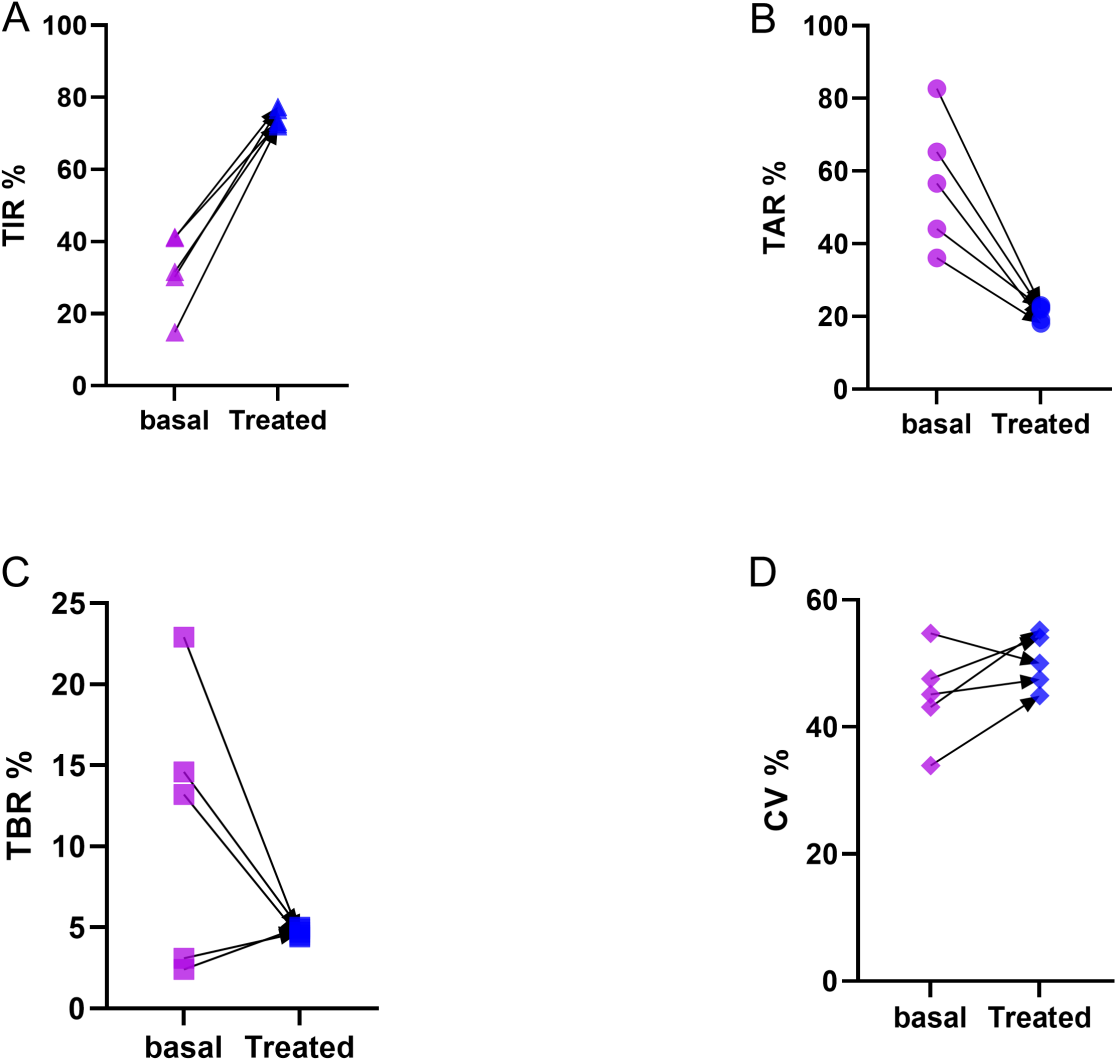
Continuous glucose monitoring (CGM) metrics before and after treatment. DATA ARE PRESENTED FOR DESCRIPTIVE PURPOSES ONLY; NO INFERENTIAL STATISTICAL TESTS WERE PERFORMED. Panels show: **(A)** Time above range (TAR, >10.0 mmol/L), **(B)** Time in range (TIR, 3.9–10.0 mmol/L), **(C)** Time below range (TBR, <3.9 mmol/L), and **(D)** Coefficient of variation (CV) in five patients during basal period and after treatment.

The successful implementation of MMF therapy in this refractory case underscores the potential of immunosuppressive strategies in severe pediatric EIAS. The detailed therapeutic protocol is outlined in **Table 2**, and the glycemic outcomes are illustrated in **Figure 2**. **Table 3** provides a comprehensive summary of the clinical characteristics, management strategies, and outcomes for all five patients.

**Table 2:** Therapeutic plan with mycophenolate mofetil.

Component	Details
Starting Dose	150 mg twice daily (escalated to 600–1,000 mg/m²/day)
Dose Escalation	Increase to 1,000 mg/m²/day after 2 weeks, under close monitoring
Insulin Regimen	Continue basal insulin degludec plus prandial insulin aspart; doses adjusted dynamically according to CGM data
Safety Monitoring	Complete blood count (CBC), liver and renal panels every 2–4 weeks; immunoglobulin levels every 3 months. No clinically significant bone−marrow suppression, hepatic/renal toxicity, or severe infections were observed. Mild gastrointestinal discomfort resolved spontaneously.

**Table 3:** Clinical characteristics, management, and outcomes of pediatric T1DM patients with EIAS.

Case	Age/Sex	Key clinical characteristics	Key laboratory findings	Intervention	Discharge regimen	HbA1c at 3 months post-optimization (mmol/mol [%])
1	6 yr 11 mo F	DKA on 21 Oct 2022 (pH 7.10, glucose 28 mmol/L); readmission 1 Oct 2023 with marked glucose variability, recurrent hypoglycaemia, IA 1.58 COI	IA 1.58 COI (positive)	Switched to ultra−rapid insulin + closed−loop pump (6−h active insulin) Basal 14.2 U/24 h; Bolus 6 U (B), 5 U (L), 6 U (D)	Pre−prandial 4–9 mmol/L, post−prandial 5.4–7.7 mmol/L; no severe hypoglycemia	56 [7.3]
2	1 yr F	DKA on 10 Jan 2021 (pH 7.12, glucose 27.1 mmol/L); readmission 17 Sep 2023 with severe glycaemic swings, seizure-inducing hypoglycaemia, IA 5.22 COI	IA 5.22 COI (positive)	Sub-Q regular insulin every 4 h (started 26 Sep 2023) 4 U regular insulin q4h	Fasting 5.3–7.3 mmol/L, post−prandial 7.2–11.9 mmol/L; severe hypoglycemia eliminated	58 [7.5]
3	4 yr 11 mo M	December 2022 diagnosed with type 1 DM. DKA on 31 Aug 2024 (pH 7.13, glucose 27.9 mmol/L); IA 2.66 COI; poor control on pump (daytime hyperglycaemia, nocturnal hypoglycaemia)	IA 2.66 COI (positive)	Switched from pump to conventional MDI (4–6 injections, total 0.875 U/kg) 4–6 daily injections	No severe hypoglycemia after change	41 [5.9]
4	3 yr 1 mo M	First admission 2 Apr 2020 (DKA, pH 7.09, glucose 26.7 mmol/L, GAD-65 14.55 IU/mL, IA 0.16 COI); multiple relapses; poor control despite various regimens	IA 2.16 COI (positive)	4–6 daily rapid−acting aspart + once−daily glargine (total ≈0.92 U/kg) 26 U total at discharge (≈0.92 U/kg)	Stable control, no severe hypoglycemia	61 [7.7]
5	8 mo M	Diagnosed 8 mo 2021; persistent glucose 2.7–15.8 mmol/L, low C-peptide, IA > 25 COI, insulin levels > 600 pmol/L, CV 43.5 % on CGM; frequent severe hypo-/hyper-glycaemia and ketoacidosis	IA 33.4 COI (positive)	MMF (150 mg twice daily) + tailored insulin regimen (degludec + aspart)	TIR > 70%, TBR < 5%, no severe hypoglycemia	62 [7.8]

EIAS, exogenous insulin antibody syndrome; T1DM, type 1 diabetes mellitus; DKA, diabetic ketoacidosis; NPH, neutral protamine hagedorn (intermediate-acting insulin); Aspart, rapid-acting insulin analogue; Detemir/Glargine/Degludec, long-acting or ultra-long-acting insulin analogues; Mycophenolate mofetil, MMF; time in range, TIR; time below range, TBR.

## 4 Literature review

**Figure 3:**
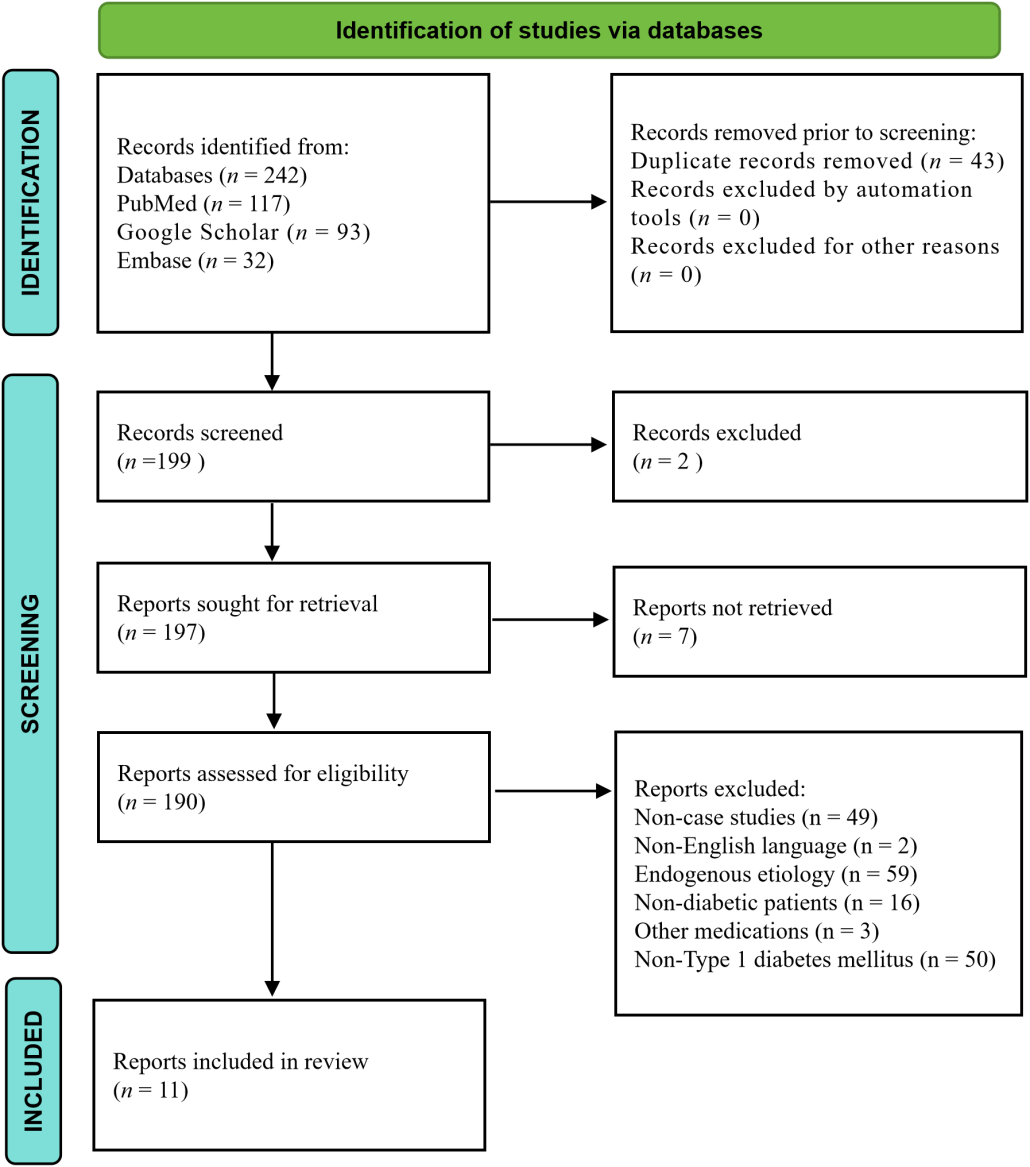
PRISMA 2020 flow diagram of literature search and selection process. Database searching of PubMed, Google Scholar, and Embase yielded 242 records. After duplicate removal (n = 43) and title/abstract screening (n = 2 excluded), 197 full-text articles were assessed; 7 were unavailable and 179 were excluded [non-case study design (n = 49), endogenous etiology (n = 59), non-Type 1 diabetes (n = 50), non-diabetic population (n = 16), non-English (n = 2), other medication causes (n = 3)], leaving 11 reports in the final review.

To contextualize our findings within the broader literature, we conducted a comprehensive review of published EIAS cases in T1DM patients. A comprehensive literature search was conducted across PubMed, Embase, and Google Scholar from January 1, 1976, to January 1, 2026. The search strategy employed the following terms: ((exogenous insulin antibody syndrome[Title/Abstract]) OR (insulin antibody syndrome[Title/Abstract])) OR (exogenous insulin AND autoimmune hypoglycemia). Two independent reviewers (SG and XYS) performed the literature screening, with disagreements resolved through consensus. The literature search identified 242 records across three electronic databases (PubMed, Google Scholar, and Embase). After removing 43 duplicate records, 199 records underwent title and abstract screening, resulting in 2 exclusions. Of the 197 reports sought for full-text retrieval, 7 were unavailable. The remaining 190 reports were assessed for eligibility, with 179 excluded for the following reasons: non-case study design (n=49), non-English language (n=2), endogenous etiology (n=59), non-diabetic patient population (n=16), other medication-related causes (n=3), and Non-Type 1 diabetes mellitus (n=50). Ultimately, 11 reports met inclusion criteria and were included in the final narrative review. The PRISMA flow diagram is presented in **Figure 3** (16).

A comprehensive summary of previously reported cases, including patient profiles, key laboratory findings, diagnostic criteria, treatment protocols, and clinical outcomes, is provided in **Table 4**.

**Table 4:** Summary of clinical characteristics of reported cases related to EIAS in type 1 diabetes in previous publications.

Case Source [study type]	Patient profile	Patients with T1DM	Key lab findings	Diagnosis criteria	Treatment protocol	Outcome, prognosis and notes
Cunningham et al. (2026) (17) [Case Report]	8-year-old Female; New-onset T1DM; Concurrent celiac disease; Developed EIAS 2 months post-T1DM diagnosis; Later developed SIR at age 13	1	IA: 330 U/mL (ref <0.4, typically <35 in T1D); PEG recovery: 42% (low, high bound insulin); Two IA populations on Scatchard analysis (high-affinity, low-affinity); Undetectable C-peptide	EIAS: Severe glycemic variability (daytime hyperglycemia + prolonged nocturnal hypoglycemia); High IA with altered binding characteristics; Abnormal insulin clearance; SIR: Severe SC insulin resistance with normal IV sensitivity; No glycemic effect from high-dose SC insulin (≤5 U/kg/d)	**EIAS phase:** Rituximab 375 mg/m² weekly ×4 weeks (after failed steroids/IVIG); **SIR phase:** Heparinized insulin lispro (1 mL 5000U heparin + 10 mL 100U insulin lispro) via SC pump	EIAS: Success with rituximab. IA decreased; hypoglycemia improved; resumed SC pump with HbA1c 55 mmol/mol (7.2%), TIR 56%. SIR: IV insulin dependent for 14 months (HbA1c 52 mmol/mol, TIR 79%) but complicated by sepsis/line blockages; heparinized SC insulin successful for 3+ years (now age 17, HbA1c 79 mmol/mol, TIR 59%). First report of sequential EIAS followed by SIR.
Rajamohan et al. (2025) (18) [Case Report]	80-year-old Female; T1DM (5 years); Concurrent cirrhosis, portal hypertension, ascites, exocrine pancreatic insufficiency, MAFLD; Frail, hemodynamically unstable; 20 kg weight loss	1	Insulin antibodies: 6758 U/mL (ref <0.4); GAD-65: 747 U/mL (ref <5); Undetectable C-peptide; HbA1c: 8.3% (67 mmol/mol); PEG recovery: low; Insulin binding capacity assay: abnormal	Extreme insulin resistance (>300 U/d, peak 800 U/d); Refractory hyperglycemia and DKA despite high-dose IV and SC insulin; Very high insulin antibody titer (>5× ULN); Exclusion of infection, Cushing’s, lipohypertrophy	High-dose IV methylprednisolone 1 mg/kg ×5 days; MMF 500 mg bid → 1 g bid; Tapering oral prednisone 50 mg by 10 mg q2w. Switched to insulin lispro	Success. Insulin req. dropped from 762 U/d to 44 U/d within 48 hours; 8-fold reduction in insulin antibodies (6758 → 842 U/mL); Remission maintained >2 years on 70 U/d insulin and 5 mg prednisone; Died of buccal SCC at 27 months. Plasmapheresis deferred due to frailty. First case using high-dose steroids as primary therapy without plasmapheresis/rituximab.
Saba et al. (2024) (4) [Case Report]	17-year-old Caucasian Male; T1DM (8 years, diagnosed age 9); Normal weight; Abrupt insulin requirement increase	1	Total insulin: 7 µIU/mL; Free insulin: 4.8 µIU/mL (68% of total); Post-treatment: Total 5.2, Free 4.6 µIU/mL (88% free)	Insulin requirement >2 U/kg/day without obesity; Low free insulin ratio (<70%); Elevated total insulin with disproportionately high bound insulin; Failed insulin analog switch	Mycophenolate Mofetil (MMF) Oral monotherapy (dose not specified)	Success. Insulin req. decreased from 3.3 to 1.4 U/kg/d (58% reduction) after 7 months; Free insulin ratio normalized from 68% to 88%; No adverse effects; Outpatient setting; First pediatric case of MMF monotherapy for EIAS.
Jerkins et al. (2024) (2) [Case Report]	84-year-old Female; Long-standing T1DM (34 years); Complicated by retinopathy, CKD 3A, and peripheral vascular disease; Suspected COVID-19 related autoimmunity	1	Insulin antibodies: 22 μU/mL (normal <4 μU/mL, >5× ULN); GAD-65: 25.2 U/mL (normal <5); Undetectable C-peptide; HbA1c: 10.1%; Fructosamine: 501 μmol/L (normal 0–285)	Severe insulin resistance; Highly positive insulin antibody titer (>5× ULN); Recurrent hyperglycemia and hypoglycemia despite high-dose insulin; Normal response to IV insulin; DKA after 34 years of T1DM	Mycophenolate Mofetil (MMF) 500 mg bid; CSII with human regular insulin; Low-cost outpatient approach	Success. Insulin req. decreased from 89 U/d to 50 U/d. Time in range improved from 9% to 55%. HbA1c improved to 6.7%. Insulin antibodies declined by 18% at 2 weeks. First report of DKA accompanying EIAS treatment. Planned 6-month MMF course.
Liu et al. (2023) (19) [Case Series]	23 patients (9 T1DM, 14 T2DM); Mean age 50 years (range 9–79); Female 65.2%; Mean BMI 22.89 kg/m²; Mean diabetes duration 9 years; 21.7% autoimmune comorbidities; 48% food/drug allergy; 30% insulin allergy; 22% lipodystrophy; 25% ANA positive	9 (39%)	IA positive >90% (mean IA/ULN ratio 90.0, range 0.4–691.5); Insulin/C-peptide molar ratio >7 in 85% (mean 22.6); PEG precipitation test: high bound insulin; HLA genotyping: DRB1*0406 in 20% (vs 90% in Hirata disease); DRB1*0405-DQB1*0401 in 50% of T1DM; DRB1*0901-DQB1*0303 in 25%	Recurrent hypoglycemia despite insulin discontinuation (T2DM); IA positivity; Elevated insulin/C-peptide molar ratio >1; PEG test showing IA-insulin complexes; Alternating hyperglycemia and hypoglycemia in >90%	Dietary intervention (all); Alpha-glucosidase inhibitors (~50%); Glucocorticoids (3 patients); Plasmapheresis (1 patient); Insulin formulation switch	Spontaneous remission in ≥69.6% (mean time 1.5 months); Favorable prognosis; IA and insulin levels decreased significantly after remission; HLA genotypes for T1DM or Hirata disease may not explain EIAS susceptibility; Largest case series (n=23) with largest T1DM cohort (n=9) reported to date.
Li et al. (2021) (5) [Case Series]	122 patients total; Median age: 67 years (range 14-86); Median diabetes duration: 10 years; Region: Mainly Asia (97.54%)	6*	Serum insulin: ≥100 U/mL in majority; C-peptide: ≤10 ng/mL (low); IA: Positive in 100%; HbA1c: 7.7% (median, range 5.4-14.4%)	Hypoglycemia + high insulin + low C-peptide + positive IA	Switched to oral hypoglycemic drugs: 55.74%; Switched to other insulin types: 27.05%; Lifestyle modification: 16.39%; Corticosteroids: 31.97%; Plasmapheresis: 3.28%	Hypoglycemia disappearance: - Corticosteroids: median 1 month (range 0.07-6); - Non-corticosteroids: median 2 months (range 0.03-8); IA turn negative: median 6 months (range 1-12); Follow-up: median 6 months (range 0.5-60), no recurrence.
Kawamura et al. (2022) (20) [Case Series]	**Case 1:** 60-year-old Male; SPIDDM; CSII user (lispro); 1.5 years post-T1DM diagnosis; No diabetic complications; **Case 2:** 70-year-old Male; Insulin-triggered T1DM; Human insulin user; Insulin allergy; Concurrent DKA	2	**Case 1:** IA 88.7%; GAD Ab 1,285 U/mL; C-peptide 0.7 ng/mL; HbA1c 6.9%; Scatchard: extremely low K1 (0.067×10^−8^ M^−^¹), high B1 (45.0×10^−8^ M); **Case 2:** IA 89% → 78.8%; C-peptide 0.2 → <0.03 ng/mL; HbA1c 12% → 8.8%; Scatchard: extremely low K1 (0.00188×10^−8^ M^−^¹), very high B1 (140×10^−8^ M)	Recurrent nocturnal hypoglycemia + daytime hyperglycemia; High IA titers; IAS-like Scatchard characteristics (low K1, high B1); Alternating glycemic pattern; Exclusion of insulinoma, tumors, counter-regulatory hormone deficiency	**Case 1:** CSII → MDI (glulisine) + α-glucosidase inhibitor + bedtime snack; No steroids; **Case 2:** CSII → MDI (aspart) without basal insulin; Anti-allergic agents; Insulin desensitization; No steroids	**Case 1:** Success. Hypoglycemia resolved within 1 week; IA decreased to 82.9% (2017), 62.1% (2018), 35.1% (2019); Scatchard normalized (B1 decreased 10-fold); Added degludec 2017; Good long-term control; **Case 2:** Success. Hypoglycemia resolved after MDI switch; Insulin allergy disappeared; IA decreased to 78.8%; Scatchard markedly improved; Long-term stability on MDI + glargine; Both: No HLA-DRB1*04:06 (unlike IAS); T1DM-susceptible HLA present; First report of CSII-induced EIAS with detailed Scatchard analysis showing correlation between antibody characteristics and clinical course.
Kong et al. (2022) (21) [Case Report]	45-year-old Male; T1DM (15 years); Long-term insulin analog use (lispro + detemir, 5 years); History of porcine insulin (2 years) then human insulin (Humulin, 10 years); No sulfhydryl drug exposure; No family history of diabetes or autoimmune disease	1	IA binding rate: 61.95% (normal <5%); Insulin >300 mU/L during hypoglycemia; C-peptide <0.02 nmol/L (undetectable); HbA1c: 9.5%; Normal thyroid and adrenal function; No pancreatic lesions on CT	Recurrent hypoglycemic coma + DKA alternating; High IA titer with high capacity/low affinity characteristics (similar to IAS); Hyperinsulinemic hypoglycemia (insulin >300, C-peptide undetectable); Exclusion of insulinoma, sulfhydryl drug exposure, endocrine disorders; Clinical pattern mimicking IAS but with exogenous insulin	Switch from insulin analog (aspart) to recombinant human insulin (Gensulin R) via pump; Oral prednisolone 10 mg tid (30 mg/day); MDI with Humulin R and Humulin N at discharge	Success. Hypoglycemia frequency markedly reduced after 1 week; Complete resolution of hypoglycemic episodes; Discharged on 30 mg prednisolone daily; IA became negative; Highlights that exogenous insulin-induced IA can mimic IAS (high capacity, low affinity); First report from China emphasizing differential diagnosis of frequent hyperinsulinemic hypoglycemia in T1DM patients on insulin analogs.
Geng et al. (2022) (22) [Case Report]	27-year-old Female; T1DM (14 years); Concurrent cyclic vomiting syndrome (CVS); Obesity (BMI 35.7); Extremely high GADA (19,384 U/mL); Menstruation-triggered vomiting; 14-year history of T1D with undetectable C-peptide	1	IAs: 21.83% (hypoglycemic period) vs 0.34% (vomiting period); Undetectable C-peptide and endogenous insulin; Exogenous insulin: 27 μU/mL during hypoglycemia; Prolonged insulin half-life: 25h; Free/bound insulin fluctuated with glucose; HbA1c: 5.9-10.9%; HPA axis activation during vomiting (ACTH 175.2 pg/mL, cortisol 28.5 μg/dL)	CVS: Rome IV criteria (stereotypic vomiting episodes, >30×/day, 2–3 days duration, asymptomatic intervals); EIAS: Recurrent severe hypoglycemia after vomiting resolution; Dynamic free/bound insulin association/dissociation; Undetectable endogenous insulin with high exogenous insulin; Insulin recycling phenomenon; Normal gastric emptying, no diabetic gastroparesis	Insulin type changes (glulisine pump); Glucocorticoids; IVIG (32.5g ×1); Plasmapheresis (5 sessions); Rituximab 750 mg/m² ×2 doses (2 weeks apart)	Success with rituximab. IAs reduced from 25% to 4%; Striking amelioration of hypoglycemia; Unexpected dramatic reduction of CVS episodes (8-month follow-up); First reported case of concurrent CVS and EIAS in T1D; Novel mechanistic insight: IAs prolong insulin half-life via “recycling” rather than simple reservoir effect; Dynamic free/total insulin measurements validated for EIAS diagnosis; Suggests CVS may be autoimmune disorder responsive to B-cell depletion.
Jerkins & Bell (2021) (23) [Case Report]	51-year-old Female; Newly diagnosed T1DM (2 weeks); Family history of T2DM and autoimmune thyroid disease	1	Insulin antibodies: 13 U/ml (normal <5 U/ml) → <0.15 U/ml; GAD-65: 3184.1 U/ml → negative; HbA1c: 11.8%	Severe insulin resistance (>400 U/d, 5.5 U/kg); High insulin antibody titer; Recurrent DKA despite high-dose insulin; Positive islet autoantibodies	Mycophenolate Mofetil (MMF) Oral monotherapy 500 mg bid → 1000 mg bid; Planned rituximab delayed and ultimately not needed	Success. Insulin req. dropped from 400 U/d to 40 U/d (90% reduction) after 5 months. Insulin antibodies normalized. HbA1c improved to 6.7%. Prolonged remission maintained at 1 year. First reported adult case cured with MMF monotherapy, avoiding IV immunosuppressant costs.
Robbins et al. (2020) (24) [Case Report]	32-year-old Male; T1DM; Newly diagnosed CVID (low IgG, IgM, undetectable IgA, IgE; low vaccine titers); Recurrent sinopulmonary infections; Lipohypertrophy (pump removed 6 weeks prior)	1	Anti-insulin antibodies: 11.1 U/mL (ref 0.0-0.4); Elevated random insulin 27 uIU/mL with glucose 48 mg/dL; Undetectable C-peptide; Normal IGF-2; Negative sulfonylurea screen; Normal AM cortisol	Profound hypoglycemia despite insulin discontinuation (4 days); Hyperinsulinemic hypoglycemia with undetectable C-peptide; Exclusion of insulinoma, surreptitious insulin, adrenal insufficiency, non-islet cell tumor; Glucose lability (15–567 mg/dL); Failed initial immunomodulation	MMF + prednisone (initial, ineffective); TPE ×5 exchanges (1.0 plasma volume, 5% albumin, days 8, 9, 11, 12, 14); IVIG ×2 courses post-TPE; Continuous 10% dextrose infusion (days 4-13); Insulin regimen changes (glargine/aspart); Dietary modifications (20g carb limit)	Success. TPE resulted in normalization of glycemia and reduced lability; Anti-insulin antibodies decreased to 1.0 U/mL; Cessation of dextrose infusion; Discharged on day 19 on insulin pump + MMF + prednisone; Initial stabilization with hybrid closed-loop pump; Relapse after IVIG denied by insurer; Readmission with severe hypoglycemia (20-30s); Improved with IVIG; Maintenance with weekly SC immunoglobulin. First report of EIAS in CVID setting; TPE effective when immunomodulation failed.

*No specific information was provided for pediatric patients.

MMF, Mycophenolate mofetil;TPE, Therapeutic plasma exchange;IVIG, Intravenous immunoglobulin;CSII, Continuous subcutaneous insulin infusion;MDI, Multiple daily injections;IA, Insulin antibodies;IAb, Insulin antibody;GAD-65, Glutamic acid decarboxylase antibody;PEG, Polyethylene glycol;TIR, Time in range;HbA1c, Glycated hemoglobin;DKA, Diabetic ketoacidosis;SC, Subcutaneous;IV, Intravenous;SIR, Severe insulin resistance;CVS, Cyclic vomiting syndrome;CVID, Common variable immunodeficiency;SPIDDM, Slowly progressive insulin-dependent diabetes mellitus;IAS, Insulin autoimmune syndrome (Hirata disease);T2DM, Type 2 diabetes mellitus;BMI, Body mass index;ANA, Antinuclear antibodies;HLA, Human leukocyte antigen;HPA, Hypothalamic-pituitary-adrenal;IGF, Insulin-like growth factor;ACTH, Adrenocorticotropic hormone;CRP, C-reactive protein;GH, Growth hormone;MAFLD, Metabolic-associated fatty liver disease;CKD, Chronic kidney disease.

Based on the compiled case series, several key observations emerge regarding EIAS in type 1 diabetes mellitus. First, EIAS represents a rare but clinically significant complication characterized by severe glycemic lability, with patients exhibiting alternating hyperglycemia and hypoglycemia due to reversible insulin-antibody binding. Second, T1DM patients with EIAS demonstrate distinct immunological profiles, including persistently high GAD-65 antibody titers and specific HLA haplotypes (*DRB10405-DQB10401, DRB109:01-DQB103:03*), though these differ from the classic *DRB1* 04:06 association seen in insulin autoimmune syndrome. Third, therapeutic strategies have evolved from high-dose corticosteroids and plasmapheresis toward B-cell depletion therapy with rituximab and oral immunosuppression with mycophenolate mofetil, with MMF monotherapy demonstrating comparable efficacy to combination regimens in selected cases. Fourth, the prognosis appears favorable with appropriate immunomodulation, with most patients achieving sustained remission and significant reductions in insulin requirements.

Notably, our study documents EIAS in an 8-month-old infant (Case 5)—the youngest reported case to date, substantially younger than the previously reported youngest patient (8 years of age). This finding challenges assumptions regarding age-related susceptibility to insulin antibody formation, demonstrating that EIAS can occur even in infantile diabetes and significantly expanding the recognized age spectrum of this syndrome in pediatric populations.

## 5 Discussion

This retrospective case series describes the clinical characteristics and treatment outcomes of five pediatric patients with T1DM complicated by EIAS. It is important to emphasize that due to the observational nature, small sample size (n=5), and absence of a control group, any observed associations between interventions and outcomes should be interpreted with caution and cannot establish causality. These findings are hypothesis-generating and require validation in larger, prospective studies.

### 5.1 Clinical features and diagnosis of EIAS

The core clinical feature of EIAS is the extreme duality of blood glucose—alternating episodes of unpredictable severe hypoglycemia and refractory hyperglycemia. This unique glucose pattern is key to distinguishing it from other causes of blood glucose fluctuations (such as the dawn phenomenon or Somogyi effect). All five pediatric patients in our study exhibited this classic feature, with blood glucose levels fluctuating within an extremely wide range.

For diagnosis, beyond clinical presentation, high-titer IA testing serves as the laboratory gold standard (3, 25, 26). Notably, IA assays routinely used for T1DM autoimmunity diagnosis may cross-react with exogenous insulin antibodies (6). However, specific methods to distinguish endogenous from exogenous antibodies are currently lacking, necessitating comprehensive clinical history assessment for definitive judgment (27). CGM and insulin/C-peptide release testing played a crucial role in this study. They not only visually demonstrated the severity and pattern of blood glucose fluctuations, along with the metabolic status of endogenous versus exogenous insulin, providing strong diagnostic evidence, but also offered objective, continuous data support for treatment plan adjustments and efficacy assessment.

Case 5 illustrates the diagnostic challenges in very young children with diabetes, particularly in resource-limited rural settings where delayed medical access is common. Although this patient was formally diagnosed at 8 months of age, retrospective symptom history suggested onset prior to 6 months, meeting the temporal criterion for NDM. The absence of pathogenic variants in *KCNJ11, ABCC8*, *INS*, and other monogenic diabetes genes, combined with positive islet autoantibodies and subsequent development of severe EIAS, confirmed autoimmune T1DM with unusually early onset rather than monogenic NDM. This case highlights that chronological age at first clinical presentation may overestimate true disease onset in underserved populations, and that comprehensive genetic testing remains essential for accurate classification of diabetes in infancy.

### 5.2 Dynamic antibody kinetics

Contrary to the assumption that EIAS represents a static complication of T1DM, our longitudinal data reveal dynamic, fluctuating IA titers in all patients. The temporal evolution observed—ranging from initial seroconversion (Cases 1, 3) to progressive titer escalation (Cases 2, 4) and subsequent decline with immunosuppression (Case 5)—suggests that EIAS is an active, evolving immune process rather than an irreversible pharmacological complication.

The marked rise in IA titers between T1DM onset and EIAS diagnosis (median interval 2.2 years) indicates a priming period during which continuous exogenous insulin exposure drives affinity maturation and isotype switching of insulin antibodies. This is particularly evident in Case 4, where the 13.5-fold increase in IA (0.16 to 2.16 COI) over 5 years coincided with escalating glycemic instability. The subsequent fluctuation in Case 4 (decline at 6 months, rebound at 1 year) suggests that antibody titers correlate with disease activity and may serve as biomarkers for treatment response.

Importantly, the absence of thyroid autoantibodies (TPOAb, TgAb) and normal cytokine profiles in most cases during active EIAS phases supports the specificity of insulin antibody-driven pathophysiology, distinguishing EIAS from polyendocrine autoimmune syndromes or systemic immune dysregulation.

### 5.3 Fine-tuning insulin regimens as foundational therapy

The fundamental issue in EIAS lies in insulin bioavailability being severely disrupted by antibodies. Therefore, the primary therapeutic goal is to bypass or mitigate this interference. Our research indicates that for the majority of children with EIAS (4/5), fine-tuning insulin regimens represents an effective first-line treatment strategy (15). The core principle is “flattening peaks and filling valleys through frequent, low-dose administration,” avoiding the formation of high-concentration insulin “reservoirs” under the skin from single large injections, thereby reducing opportunities for antibody binding (28). Used infusion times for large doses (square wave/dual wave) essentially simulates a smoother insulin absorption curve—a key technique for controlling EIAS blood glucose fluctuations (29). In this study, four pediatric patients successfully achieved TIR above 70% using this approach, demonstrating the strategy’s efficacy. This suggests clinicians encountering suspected EIAS cases should prioritize extreme optimization of insulin delivery pharmacokinetics as a primary intervention.

It is important to note that HbA1c reflects average glucose over 2–3 months. In Case 1, the baseline HbA1c of 43.2 mmol/mol (6.1%) reflected glycemic control prior to EIAS onset, while the 3-month value of 56 mmol/mol (7.3%) captured the transition period including EIAS diagnosis and treatment adjustment. The improvement in glycemic stability is better reflected by CGM metrics (TIR improved from 41.3% to 77.4%) rather than HbA1c changes.

### 5.4 Persistent elevation of coefficient of variation despite glycemic improvement

Notably, although individualized insulin optimizations improved TIR and reduced severe hypoglycemic episodes in Cases 1–4, the coefficient of variation (CV) remained elevated (~50%) and failed to reach the target threshold of <36% (**Figure 2D**). This observation carries significant clinical implications. According to current CGM consensus, CV >36% indicates high glycemic variability and is associated with an increased frequency of hypoglycemia (30).

The persistently elevated CV despite pharmacokinetic adjustments underscores a fundamental limitation of insulin regimen modifications in EIAS management. As described in the pathophysiology of insulin autoimmune syndrome, insulin antibodies possess high binding capacity but low affinity, forming unstable insulin-antibody complexes that intermittently dissociate and release free insulin in an unpredictable manner (31). Current strategies—including switching to ultra-rapid-acting insulin, increasing injection frequency, and employing extended/square-wave boluses—primarily function by bypassing the antibody reservoir effect. By delivering smaller, more frequent doses or prolonging infusion duration, these approaches minimize the formation of high-concentration insulin depots in subcutaneous tissue, thereby reducing the probability of massive antibody binding and subsequent sudden dissociation that causes severe hypoglycemia (23).

However, these pharmacokinetic manipulations do not alter the underlying immunopathology: the high-titer insulin antibodies persist and continue to intermittently bind and release circulating insulin (6). Consequently, while extreme hyperglycemia and severe hypoglycemia are mitigated through meticulous dose adjustments, the buffering and intermittent release effect of the antibodies maintains substantial glucose fluctuations, manifesting as persistently elevated CV (6). This phenomenon is consistent with previous reports demonstrating that IA-positive patients exhibit significantly larger within-day glucose fluctuations compared to IA-negative patients (6).

In contrast, Case 5 demonstrated that immunosuppressive therapy with MMF, which directly targets antibody-producing B cells and reduces IA titers by >50%, addresses the root pathological mechanism. By fundamentally decreasing antibody production rather than merely circumventing its effects, MMF therapy offers the potential to normalize not only TIR but also glycemic variability. This distinction highlights the necessity of stratified treatment: while insulin optimization provides symptomatic control for most patients, refractory cases with persistently high CV despite regimen adjustments may require immunosuppressive intervention to achieve true metabolic stability.

Clinicians should recognize that normalized TIR with persistently elevated CV does not equate to complete glycemic stability. Patients remain at risk for unpredictable glucose excursions and should continue intensive CGM monitoring. The failure of CV normalization with insulin adjustments alone further supports the rationale for immunosuppressive therapy in antibody-mediated glycemic dysregulation, as only reduction of antibody burden can address the intermittent insulin release driving residual variability.

### 5.5 MMF offers a new treatment option for refractory EIAS

For refractory EIAS cases with extremely high antibody titers and potent affinity where conventional insulin regimens fail, direct immunomodulation to suppress antibody production represents a necessary therapeutic escalation (2, 5). Historical approaches have included potent immunosuppressants—such as glucocorticoids and cyclophosphamide—as well as plasma exchange (4, 5, 23, 32). However, their application in pediatric patients is limited due to significant side effects (5). However, these interventions present substantial limitations in pediatric populations. Corticosteroids, while effective in rapidly suppressing antibody production, induce significant metabolic adverse effects including hyperglycemia, growth retardation, and systemic immunosuppression that are particularly problematic in children with diabetes. Rituximab, a B-cell depleting agent demonstrating efficacy in adult EIAS cases (22), requires intravenous administration, incurs substantial costs, and poses risks of prolonged hypogammaglobulinemia, limiting its practicality in pediatric settings. Plasma exchange offers only transient antibody reduction without addressing underlying production, necessitating specialized facilities and repeated procedures.

Mycophenolate mofetil emerges as a favorable alternative for refractory pediatric EIAS. As a selective inhibitor of lymphocyte proliferation, MMF offers distinct advantages: convenient oral administration, relatively specific targeting of B-cell antibody production, and a milder adverse effect profile characterized by manageable myelosuppression and limited hepatic/renal toxicity compared with conventional immunosuppressants (7, 8). These pharmacological features have increasingly positioned MMF as a viable therapeutic option for pediatric autoimmune conditions.

The selection of MMF as second-line therapy should be individualized based on disease severity, antibody characteristics, patient age, comorbidities, and resource availability. Specifically, MMF is preferred for its facilitation of outpatient management and minimized systemic immunosuppression, whereas rituximab may be indicated for patients requiring rapid antibody reduction, corticosteroids for short-term bridge therapy in acute crises, and cyclophosphamide for EIAS concomitant with other severe autoimmune manifestations.

In the present study, Case 5 demonstrated significant reduction in insulin antibody titers and fundamental improvement in glycemic control following six months of MMF treatment, without severe adverse reactions. This successful case supports MMF’s potential as an effective and safe second-line therapy for refractory pediatric EIAS. By inhibiting B-lymphocyte proliferation and reducing pathogenic insulin antibody production at its source (33). MMF offers a targeted approach to this challenging clinical condition. Although this represents a single-case report, cumulative evidence from sporadic successful cases in the literature suggests that MMF provides a promising therapeutic strategy for refractory EIAS in pediatric patients.

### 5.6 Genetic heterogeneity

Case 5 exhibited several distinctive features that warrant separate consideration. Unlike the other four patients, this child harbored concurrent chromosomal abnormalities: a 47,XXY karyotype (Klinefelter syndrome) and a *de novo* Xp22.33-q28 duplication. These genetic factors may have contributed to his exceptionally severe EIAS phenotype, characterized by: (i) markedly elevated IA titers (33.40 COI vs. 1.58-5.22 COI in other cases); (ii) refractory glycemic instability unresponsive to intensive insulin optimization; (iii) requirement for immunosuppressive therapy to achieve glycemic control.

The Xp22.33-q28 region contains multiple genes involved in immune regulation and B-cell development (34, 35). Its duplication, combined with the X-chromosome polysomy inherent to Klinefelter syndrome, may have created a permissive immunological environment for high-affinity, high-titer insulin antibody production. This suggests that Case 5 may represent a genetically predisposed subtype of EIAS with distinct pathophysiological mechanisms requiring more aggressive therapeutic intervention. Clinicians should consider chromosomal microarray analysis in infantile-onset diabetes with severe, refractory EIAS to identify potential underlying genetic susceptibility factors.

### 5.7 Limitations

This study has several important limitations that significantly affect the interpretation of findings:

1. Sample size (n=5): The statistical power is extremely limited. We cannot detect rare adverse events or small effect sizes. The observed associations may be due to chance.
2. Selection bias: As a single-center retrospective study, we may have preferentially included patients with more severe disease or those who had suboptimal responses to initial treatments. This limits generalizability to the broader population of pediatric T1DM patients with EIAS.
3. Residual confounding: Unmeasured confounding factors that we could not control for include genetic background and HLA haplotypes, adherence to treatment regimens, dietary patterns and physical activity, concurrent illnesses or stressors, and healthcare access and socioeconomic factors.
4. Lack of external validation: The findings have not been validated in other centers or populations. The reproducibility of our treatment protocols in different healthcare settings is unknown.
5. Absence of control group: Without a control group receiving standard care or placebo, we cannot distinguish treatment effects from disease natural course, regression to the mean, or concurrent treatments.
6. Retrospective design: The retrospective nature introduces information bias in data collection. Not all desired measurements were available for all patients at all time points.
7. Short follow-up and case heterogeneity: The maximum follow-up was 1 year for MMF treatment; long-term efficacy and safety are unknown. Case 5’s genetic abnormalities may represent a distinct subtype, potentially limiting direct comparison with other cases.

Given these substantial limitations, the results of this study should be interpreted with extreme caution. Clinicians should not base treatment decisions solely on these findings. Instead, this study should be viewed as generating hypotheses for future prospective, controlled investigations. The treatment protocols described require validation in larger, multicenter studies before widespread clinical adoption.

## 6 Conclusion

EIAS represents a significant cause of severe glycemic dysregulation in children with T1DM, characterized by dramatic glucose fluctuations between hyperglycemia and hypoglycemia. CGM metrics (TIR, TBR, CV) are essential for assessment, as HbA1c may not adequately reflect glycemic variability.

Individualized insulin regimen adjustments are associated with improved TIR in most patients, though CV may remain elevated, indicating persistent underlying glycemic variability. For refractory cases with high IA titers and persistent glycemic instability despite insulin optimization, MMF may offer a potential therapeutic option by reducing antibody production at the source. However, this observation is based on a single case and requires validation in larger studies. Due to the observational nature, small sample size (n=5), and absence of a control group, these findings should be considered hypothesis-generating and require validation in prospective, multicenter, controlled studies. Future research should focus on establishing standardized diagnostic criteria, identifying biomarkers for treatment response, and conducting randomized trials to evaluate optimal immunosuppressive strategies. Clinicians should enhance awareness of EIAS to enable timely diagnosis and implementation of appropriate individualized therapeutic measures.

## Data Availability

The original contributions presented in the study are included in the article/**Supplementary Material**. Further inquiries can be directed to the corresponding author/s.

## References

[1] Riaz M Akram M Ibrahim MN Khoso ZA . Frequency of C-peptide and antibody levels (anti GAD, Islet cell antibodies, insulin auto antibodies) in children of Pakistan with Type-1 diabetes. Pak J Med Sci. (2024) 40:1083–6. doi: 10.12669/pjms.40.6.7794, PMID: 38952492 PMC11190383

[2] Jerkins T Stockham K Bell DSH . Exogenous insulin antibody syndrome (EIAS) presenting in an elderly, long-term patient with type 1 diabetes mellitus that resolved with low-cost outpatient therapy with mycophenolate mofetil and regular insulin by pump. Diabetes Ther: Res Treat Educ Diabetes Relat Disord. (2024) 15:1473–81. doi: 10.1007/s13300-024-01573-y, PMID: 38653902 PMC11096292

[3] Quan H Tan H Li Q Li J Li S . Immunological hypoglycemia associated with insulin antibodies induced by exogenous insulin in 11 chinese patients with diabetes. J Diabetes Res. (2015) 2015:746271. doi: 10.1155/2015/746271, PMID: 25961056 PMC4413037

[4] Saba L Fatica EM Orandi AB Pittock ST Creo AL . Exogenous insulin antibody syndrome in a pediatric patient: successful treatment with mycophenolate mofetil. Horm Res Paediatr. (2024) 97:303–10. doi: 10.1159/000531767, PMID: 37660694

[5] Li Z Yi D Zheng L Li S Fang W Wang C . Analysis of the clinical characteristics of insulin autoimmune syndrome induced by exogenous insulin in diabetic patients. Diabetol Metab Syndr. (2021) 13:38. doi: 10.1186/s13098-021-00658-z, PMID: 33827670 PMC8028117

[6] Hu X Chen F . Exogenous insulin antibody syndrome (EIAS): a clinical syndrome associated with insulin antibodies induced by exogenous insulin in diabetic patients. Endocr Connect. (2018) 7:R47–55. doi: 10.1530/EC-17-0309, PMID: 29233817 PMC5776673

[7] Downing HJ Pirmohamed M Beresford MW Smyth RL . Paediatric use of mycophenolate mofetil. Br J Clin Pharmacol. (2013) 75:45–59. doi: 10.1111/j.1365-2125.2012.04305.x, PMID: 22519685 PMC3555046

[8] Ehren R Schijvens AM Hackl A Schreuder MF Weber LT . Therapeutic drug monitoring of mycophenolate mofetil in pediatric patients: novel techniques and current opinion. Expert Opin Drug Metab Toxicol. (2021) 17:201–13. doi: 10.1080/17425255.2021.1843633, PMID: 33107768

[9] Agha RA Fowler AJ Lee S-Y Gundogan B Whitehurst K Sagoo HK . Systematic review of the methodological and reporting quality of case series in surgery. Br J Surg. (2016) 103:1253–8. doi: 10.1002/bjs.10235, PMID: 27511619

[10] Haller MJ Bell KJ Besser REJ Casteels K Couper JJ Craig ME . ISPAD clinical practice consensus guidelines 2024: screening, staging, and strategies to preserve beta-cell function in children and adolescents with type 1 diabetes. Horm Res Paediatr. (2024) 97:529–45. doi: 10.1159/000543035, PMID: 39662065 PMC11854978

[11] Battelino T Danne T Bergenstal RM Amiel SA Beck R Biester T . Clinical targets for continuous glucose monitoring data interpretation: recommendations from the international consensus on time in range. Diabetes Care. (2019) 42:1593–603. doi: 10.2337/dci19-0028, PMID: 31177185 PMC6973648

[12] Fujita N Yamamoto Y Hayashino Y Kuwata H Okamura S Iburi T . Real-life glycemic control in patients with type 2 diabetes treated with insulin therapy: A prospective, longitudinal cohort study (Diabetes Distress and Care Registry at Tenri [DDCRT 9]). J Diabetes Investig. (2018) 9:294–302. doi: 10.1111/jdi.12693, PMID: 28494126 PMC5835478

[13] Giugliano D Maiorino MI Bellastella G Chiodini P Ceriello A Esposito K . Efficacy of insulin analogs in achieving the hemoglobin A1c target of <7% in type 2 diabetes: meta-analysis of randomized controlled trials. Diabetes Care. (2011) 34:510–7. doi: 10.2337/dc10-1710, PMID: 21216850 PMC3024378

[14] Alotaibi A Al Khalifah R McAssey K . The efficacy and safety of insulin pump therapy with predictive low glucose suspend feature in decreasing hypoglycemia in children with type 1 diabetes mellitus: A systematic review and meta-analysis. Pediatr Diabetes. (2020) 21:1256–67. doi: 10.1111/pedi.13088, PMID: 32738022

[15] Ishii T Katayama A Sue M Kuribayashi R Tenta M Matsushita Y . Case of subcutaneous insulin resistance syndrome treated with ultra-rapid insulin lispro. J Diabetes Investig. (2022) 13:588–91. doi: 10.1111/jdi.13667, PMID: 34510782 PMC8902381

[16] Page MJ McKenzie JE Bossuyt PM Boutron I Hoffmann TC Mulrow CD . The PRISMA 2020 statement: an updated guideline for reporting systematic reviews. BMJ. (2021) 372:n71. doi: 10.1136/bmj.n71, PMID: 33782057 PMC8005924

[17] Cunningham V Lam L Hsiao K-C Ayers R Albert B Jefferies C . Exogenous insulin antibody syndrome and subsequent severe subcutaneous insulin resistance complicating type 1 diabetes. JCEM Case Rep. (2026) 4:luaf300. doi: 10.1210/jcemcr/luaf300, PMID: 41472947 PMC12747807

[18] Rajamohan HH Boyer P-N Davis T Lane M Love A . Exogenous insulin antibody syndrome: A rare cause of extreme insulin resistance treated with high-dose corticosteroids. JCEM Case Rep. (2025) 3:luaf175. doi: 10.1210/jcemcr/luaf175, PMID: 40860576 PMC12371233

[19] Liu Y Ping F Yu J Lv L Zhao Y Qi M . Hypoglycemia caused by exogenous insulin antibody syndrome: A large single-center case series from China. J Clin Endocrinol Metab. (2023) 108:713–7. doi: 10.1210/clinem/dgac578, PMID: 36219196

[20] Kawamura R Miyao S Onuma H Uchigata Y Kawasaki E Ohashi J . Recurrent hypoglycemia due to a high titer of insulin antibody in response to exogenous insulin administration in two cases of type 1 diabetes. Intern Med. (2022) 61:687–95. doi: 10.2169/internalmedicine.7647-21, PMID: 34471020 PMC8943391

[21] Kong Y Zhang Y Cheng L Ling C Hu X Kong Y . Recurrent hypoglycemic coma and diabetic ketoacidosis caused by insulin antibody. A rare case of type 1 diabetes mellitus. Rev méd Chile. (2022) 150:115–9. doi: 10.4067/S0034-98872022000100115, PMID: 35856973

[22] Geng L Diao X Han H Lin Y Liang W Xu A . Type 1 diabetes complicated with cyclic vomiting syndrome and exogenous insulin antibody syndrome: A case report. Front Endocrinol. (2022) 13:1043301. doi: 10.3389/fendo.2022.1043301, PMID: 36440205 PMC9684460

[23] Jerkins T Bell DSH . Development of exogenous insulin antibody syndrome in a patient with newly diagnosed type 1 diabetes successfully treated with oral immunosuppressive monotherapy. Diabetes Ther: Res Treat Educ Diabetes Relat Disord. (2021) 12:2795–9. doi: 10.1007/s13300-021-01129-4, PMID: 34403116 PMC8479014

[24] Robbins DJ Taylor NE Narayanan D Hess AS Rose WN . Therapeutic plasma exchange for exogenous insulin antibody syndrome in combined variable immunodeficiency: a case report. J Clin Apher. (2020) 35:128–30. doi: 10.1002/jca.21769, PMID: 31981239

[25] Liminet C Vouillarmet J Chikh K Disse E . Antibody-mediated insulin resistance: when insulin and insulin receptor act as autoantigens in humans. Can J Diabetes. (2016) 40:462–5. doi: 10.1016/j.jcjd.2016.02.007, PMID: 27062110

[26] Wasada T Sakimoto T Aso Y Kato K Ibayashi H Omori Y . A case of insulin resistant diabetes with possible antibodies to insulin receptors. Endocrinol Jpn. (1979) 26:19–26. doi: 10.1507/endocrj1954.26.19, PMID: 436798

[27] Geng L Wong C-L Liao B Lin Y Han H Lam KSL . Development of a novel diagnostic assay for insulin receptor autoantibodies based on a patient with autoimmune hypoglycaemia. Front Endocrinol (Lausanne). (2022) 13:1029297. doi: 10.3389/fendo.2022.1029297, PMID: 36387920 PMC9642853

[28] Hermansen K Fontaine P Kukolja KK Peterkova V Leth G Gall MA . Insulin analogues (insulin detemir and insulin aspart) versus traditional human insulins (NPH insulin and regular human insulin) in basal-bolus therapy for patients with type 1 diabetes. Diabetologia. (2004) 47:622–9. doi: 10.1007/s00125-004-1365-z, PMID: 15298338

[29] Dungan K Hall C Schuster D Osei K . Comparison of 3 algorithms for Basal insulin in transitioning from intravenous to subcutaneous insulin in stable patients after cardiothoracic surgery. Endocr Pract: Off J Am Coll Endocrinol Am Assoc Clin Endocrinol. (2011) 17:753–8. doi: 10.4158/EP11027.OR, PMID: 21550950 PMC3191282

[30] Urakami T Terada H Tanabe S Mine Y Aoki M Aoki R . Clinical significance of coefficient of variation in continuous glucose monitoring for glycemic management in children and adolescents with type 1 diabetes. J Diabetes Investig. (2024) 15:1669–74. doi: 10.1111/jdi.14303, PMID: 39230367 PMC11527802

[31] Lin M Chen Y Ning J . Insulin autoimmune syndrome: A systematic review. Int J Endocrinol. (2023) 2023:1225676. doi: 10.1155/2023/1225676, PMID: 36844104 PMC9946739

[32] Sweis NWG Albanna A Alhasoun R Zayed A . A possible novel effect for dapagliflozin in the management of subcutaneous insulin resistance syndrome: a report of two cases. Int J Endocrinol Metab. (2022) 20:e126350. doi: 10.5812/ijem-126350, PMID: 36407033 PMC9661541

[33] Orsini A Costagliola G Perna D Esposito MG Bonfiglio L Striano P . Efficacy and tolerability of mycophenolate mofetil in a pediatric Rasmussen syndrome. Epilepsy Behav Rep. (2020) 13:100334. doi: 10.1016/j.ebr.2019.100334, PMID: 32140679 PMC7044645

[34] Carmona FD Cénit MC Diaz-Gallo L-M Broen JCA Simeón CP Carreira PE . New insight on the Xq28 association with systemic sclerosis. Ann Rheum Dis. (2013) 72:2032–8. doi: 10.1136/annrheumdis-2012-202742, PMID: 23444193 PMC3818491

[35] Leone F Gori A Cinicola BL Brindisi G Maglione V Anania C . Extra X, extra questions: Trisomy X syndrome and IgA deficiency - a case report. Front Immunol. (2024) 15:1518076. doi: 10.3389/fimmu.2024.1518076, PMID: 39712011 PMC11659227

